# Female urinary incontinence and wellbeing: results from a multi-national survey

**DOI:** 10.1186/s12894-016-0140-z

**Published:** 2016-05-23

**Authors:** Andrew P. Smith

**Affiliations:** Centre for Occupational and Health Psychology, School of Psychology, Cardiff University, 63 Park Place, Cardiff, CF10 3AS UK

**Keywords:** Urinary incontinence, Wellbeing, Female, Quality of life, Exercise, Sex

## Abstract

**Background:**

Previous research has shown that the severity of symptoms of urinary incontinence impacts on quality of life and wellbeing.

The aim of this article is to investigate the relationship between female urinary incontinence and mental wellbeing. This involved analyses comparing those with UI and those without to determine whether any differences in wellbeing were modified by demographic factors, specific wellbeing domain, or exercise and frequency of sex. Following this, further analyses compared sub-groups of those with UI (based on the impact of the UI) to determine which characteristics were important in influencing wellbeing.

**Methods:**

An internet survey of women with UI, aged between 45 and 60 years, has been previously reported and this article reports secondary analyses of that data.

A sample from 4 countries: the UK, France, Germany and the USA.

Two thousand four hundred three women completed the survey, 1203 with UI and 1200 who did not report UI.

The main outcome measures were the scores from the Warwick-Edinburgh Mental Wellbeing Scale (WEMWBS).

**Results:**

The results showed that lower wellbeing is observed in UI. This effect is observed in all aspects of wellbeing and most sub-groups of UI sufferers. Lifestyle influences wellbeing and those with UI who exercise less frequently or have sex infrequently are especially likely to report lower wellbeing. Wellbeing decreases as a function of the indirect measures of severity of UI and reductions in HRQol. Again, these changes reflect all aspects of wellbeing measured by WEMWBS.

**Conclusions:**

The results show that women with UI, aged 45–60 years, report lower wellbeing. This effect was not modified by demographic factors and was apparent in most of the domains measured by the WEMWBS. The reduced wellbeing was related to the impact of the UI on behaviour, embarrassment associated with it, and frequency of leakage.

## Background

The prevalence of urinary incontinence (UI) in adult women has been estimated to be about 18 % [1] and in the elderly this figure can be as high as 55 % [2]. UI can negatively affect many aspects of life, and has been shown to affect overall health-related quality of life (HRQoL) [3–12]. Indeed, a search of PubMed reveals 28 articles that have examined UI and HRQol. A recent survey [13] aimed to develop a clear understanding of the impact of urinary incontinence on the quality of life (Qol) of women of working age. The results showed that the severity of symptoms of UI impacted on quality of life. The results also showed that wellbeing decreased as the symptoms of UI became more severe. The relationship between HRQol and wellbeing was also examined and it was found that UI reduced HRQol which in turn led to reduced wellbeing.

### Wellbeing

Historically, research on wellbeing has mainly been conducted within two traditions: hedonic wellbeing and eudaimonic wellbeing. The hedonic approach to wellbeing measures subjective wellbeing which is comprised of life satisfaction, positive affect, and an absence of negative affect. Specifically, focusing on happiness from the perspective of pleasure versus pain including both cognitive evaluation (life satisfaction) and affect (with positive and negative affect comprising separate dimensions). In contrast, the eudaimonic approach to wellbeing is not simply interested in subjective happiness, but in the realisation of human potential. Within this view, wellbeing is linked to a person living in a way which is congruent with their deeply held values: a meaningful life characterised by personal growth, as opposed to a pleasurable life characterised by hedonic enjoyment. There is now international interest in wellbeing and its role in all aspects of life. This interest can be seen at a population level and in specific contexts such as work. In addition, wellbeing is now an outcome of interest in many health-related areas. Wellbeing scales such as the Warwick-Edinburgh Wellbeing Scale (WEMWBS;) [14] measure both hedonic and eudaimonic wellbeing and are correlated with general health, positive and negative affect, life satisfaction and mental ill-health. UI has been shown to negatively impact on these outcomes and it is important to investigate whether wellbeing scores are related to UI as at the moment there is little information on this topic.

### Wellbeing and UI

There have been a number of studies which have investigated associations between incontinence and mental health [15–17]. These generally confirm that UI is associated with an increased risk of depression [15], and show that treatment of the depression associated with UI may be an effective way of reducing the burden of UI [16]. Indeed, interventions aimed at increasing resilience may lessen the impact of depression on those with UI [17].

Two Australian studies have investigated associations between UI and wellbeing [18, 19]. The first study [18] showed that UI was associated with reduced wellbeing but that the relationship between different types of UI and wellbeing was different. The second study [19] investigated associations between UI and wellbeing in young nulligravid women. The results confirmed that women with UI reported lower wellbeing that women without UI. It is now important to replicate and extend these findings using multi-national samples of women of working age and recently developed measures of wellbeing.

### Aims

The primary aim was to conduct secondary analyses of our previous study [13] which focused on UI in women of working age to provide a detailed profile of female UI and wellbeing. This involved comparison of the wellbeing of those with UI with those with no symptoms. Examination of the individual items of the WEMWBS was carried out to determine whether effects are global or restricted/greatest in specific domains. Further analyses examined whether the associations between UI and wellbeing (and effects on wellbeing of those without UI) varied as a function of demographics and lifestyle (frequency of exercise and sex). Our previous paper from this survey [13] focused on associations between UI severity, HRQoL and WEMBS. UI symptoms were associated with lower HRQoL, which then impacts negatively on wellbeing. In the analyses reported here sub-sets of the UI group (based on indicators of severity - e.g., frequency of wearing pads; interference with activities) were also compared. The general hypothesis being tested was that even relatively mild UI would be associated with reduced wellbeing (compared to those without UI) and that such effects would be global rather than restricted to specific domains or sub-groups. Additionally, the analyses examined whether there was variation within the UI group as a function of the impact of UI on different activities and outcomes. These secondary analyses extend the initial aims of the protocol and address issues that are of practical relevance to women of working age with UI.

## Method

The survey was conducted by Ipsos Market Research and adhered to the Market Research Society code of conduct and was carried out with the informed consent of the participants. The Ipsos samples are representative of the country in terms of demographics and are selected using the recruitment criteria of the specific survey (see below).

### Study population

An internet survey (with the questions in the appropriate language) was conducted in 4 countries (France, Germany, UK and USA) during September 2013. A sample of women aged 45–60 years was recruited by Ipsos Marketing. Their online panels currently consist of approximately 920,000 members in the USA, 535,000 in France, 300,000 in the UK, and 169,000 in Germany. The survey was answered by both women with and without UI and the sampling procedure meant that the UI and non-UI groups did not differ in terms of basic demographics. Informed consent was represented by participation in the survey after the provision of the information about the study. Participants were aware of this procedure when they joined the online panels. The first 300 respondents with UI in each of the four countries were included in the study population, as were the first 300 non-UI respondents. This procedure meant that it was impossible to determine how many would have completed the survey if there was no sample cut-off point nor how many would have refused to participate. Sample size was based on the need to detect small effect sizes (Cohen’s d = 0.2; with power set at 0.9 and *p* = 0.05 a total sample size of 1054 would be needed) in the wellbeing outcomes. Participants were paid for participating in the study and after this the database was anonymised.

### Survey questionnaire

UI categorisation was defined by the answer to the ICIQ-UI Short Form question “how often do you leak urine?; those answering “Never” were classed as without UI while all other answers were classed as having UI. UI severity was based on the response to “How much urine do you usually leak?” [20] with a small amount being classified as “light UI”, a moderate amount as “medium UI” and a large amount as “severe UI”. Those reporting UI completed questions measuring UI symptoms (ICIQ-UI short form) [21] and UI quality of life (ICIQ-LUTSqol) [20]. Both UI and non-UI participants completed the WEMWBS [14] which has 14 items and a Likert scale of 1–5 giving a maximum score of 70.

Demographic factors (e.g., age; marital status) were recorded as were frequency of exercise and sex. Other questions examined the impact of UI on aspects of life. Outcomes of UI were also recorded (degree of embarrassment; frequency of wearing pads). The complete set of questions are given in supplementary information with our previous publication from the study [13].

### Statistical analysis

The first set of analyses compared those reporting UI with those who did not. Analyses were carried out on the individual items of the wellbeing scale to determine whether any specific domains were more impaired than others. Sub-groups based on demographic factors were also compared. The initial analyses involved a MANOVA to determine whether effects were apparent across all items of the wellbeing scale. Most studies use the WEMWBS total score but analysis of the individual items provided an opportunity to examine the generality of effects. Subsequent analyses examined whether effects were apparent in sub-groups of patients (based on demographics, frequency of exercise and sex) and used univariate ANOVAs followed by multiple regression. Factor analyses were carried out to identify categories of outcomes associated with UI. ANOVAs were then carried out to examine changes in wellbeing as a function of the severity of these different types of problem. A similar approach was used to examine the effects of factors leading to leakage on wellbeing. The analyses were carried out using the IBM SPSS Statistics 20 package.

## Results

There was no missing data for the variables considered in the initial analyses comparing those reporting UI with the non-UI group. Those with UI reported significantly lower wellbeing (UI mean: 48.2 s.d. 10.2; Non-UI mean: 50.2 s.d. 8.9; t = 5.3 d.f. 2401 *p* < 0.001) and a multivariate analysis of variance showed this was significant for all items in the scale (Wilks Lambda = 0.97 *p* < 0.001; see Table 1 for means and s.d.s.

**Table 1:** Scores for individual wellbeing questions (higher scores = greater wellbeing)

Question	Group	Mean	SD	Significance
Feeling cheerful	UI	3.39	0.91	*F* = 17.0 *p* <0.001
	Non-UI	3.53	0.84	
Thinking clearly	UI	3.71	0.95	*F* = 12.2 *p* <0.001
	Non-UI	3.84	0.89	
Close to other people	UI	3.45	0.99	*F* = 17.0 *p* <0.001
	Non-UI	3.61	0.87	
Feeling confident	UI	3.35	0.97	*F* = 27.0 *p* < 0.001
	Non-UI	3.54	0.90	
Dealing with problems well	UI	3.58	0.88	*F* = 12.0 *p* < 0.001
	Non-UI	3.70	0.81	
Energy to spare	UI	2.79	1.05	*F* = 44.8 *p* < 0.001
	Non-UI	3.06	0.94	
Feeling good about myself	UI	3.35	0.96	*F* = 19.0 *p* < 0.001
	Non-UI	3.51	0.87	
Interested in new things	UI	3.46	1.00	*F* = 13.4 *p* < 0.001
	Non-UI	3.60	0.88	
Interested in other people	UI	3.56	0.98	*F* = 9.9 *p* < 0.001
	Non-UI	3.68	0.87	
Feeling loved	UI	3.56	1.09	*F* = 7.8 *p* < 0.005
	Non-UI	3.68	1.02	
Feeling optimistic about future	UI	3.25	1.06	*F* = 7.3 *p* < 0.01
	Non-UI	3.36	0.96	
Able to make up own mind	UI	4.03	0.88	*F* = 12.1 *p* < 0.005
	Non-UI	4.15	0.79	
Feeling relaxed	UI	3.21	0.95	*F* = 6.7 *p* < 0.01
	Non-UI	3.31	0.89	
Feeling useful	UI	3.46	0.98	*F* = 25.0 *p* <0.001
	Non-Ui	3.66	0.89	

All of the individual effects remained significant when a Holm-Bonferroni correction for multiple comparisons was applied. Subsequent analyses examined whether the effect varied as a function of country, age and marital status. The results (see Table 2) showed that the effect of UI was observed in the majority of sub-groups groups (the exceptions being the French and those who were widowed). There was no missing data in these analyses.

**Table 2:** Total wellbeing scores in the UI and non-UI groups as a function of country, age and marital status (high scores = greater wellbeing)

Group	Mean	SD	N
USA UI	48.2	10.9	300
USA non-UI	51.9	9.9	300
UK UI	46.1	10.2	300
UK non-UI	48.8	9.2	301
Germany UI	49.7	10.2	301
Germany non-UI	51.6	8.6	300
France UI	48.7	9.4	301
France non-UI	48.7	7.3	300
Country x UI group interaction: *F* (3, 2395) = 3.8 *p* < 0.05			
Age			
45–48 years UI	47.6	9.9	262
45–48 non-UI	49.4	9.2	292
49–53 years UI	47.4	10.3	367
49–53 non-UI	49.8	8.5	342
54–57 years UI	48.8	10.8	247
54–57 non-UI	51.2	8.8	233
58–60 years UI	49.0	10.1	327
58–60 years non-UI	50.8	9.1	333
Age x UI group interaction: *F* < 1			
Marital Status			
Single UI	47.0	9.5	164
Single non-UI	48.3	8.5	202
Married/living together UI	49.0	9.9	743
Married non-UI	51.0	8.9	695
Widowed UI	47.7	8.9	51
Widowed non-UI	46.9	10.4	49
Divorced/separated UI	46.6	11.8	245
Divorced non-UI	50.3	8.7	254
Marital status x UI group interaction: F 93,2395) = 2.1 *p* > 0.05)			

A multi-variate analysis was then carried out in which UI v non-UI, country, age, marital status, frequency of exercise and frequency of sex were entered into a regression with wellbeing as the outcome. All of the effects, except marital status, were significant (see Table 3) confirming the results of the individual analyses.

**Table 3:** Multi-variate regression with total WEMWBS score as the dependent variable

Variable	B	Std error	Standardised B	t	Sig
Age	1.557	0.359	.086	4.334	<0.001
Exercise	−1.596	.163	−.194	−9.784	<0.001
Sex	−.684	.122	−.111	−5.588	<0.001
UI v non-UI	1.886	.356	.105	5.302	<0.001

The next set of analyses examined effects of frequency of exercise (no missing data) and sex (507 participants preferred not to answer this question, with there being significantly more in this category in the non-UI group) on wellbeing in both groups. Reduced wellbeing in the UI group was greatest in those who did not frequently exercise. Frequency of having sex was associated with greater wellbeing and this was true for both UI and non-UI groups (see Table 4).

**Table 4:** Frequency of exercise and sex and wellbeing (high scores = greater wellbeing; More exercise = every day or once or twice a week; Less exercise = once or twice a month or never. More frequent sex = once or twice a week or more; Less frequent sex = once or twice a month or I can’t remember when)

Group	Mean	SD	N
UI more exercise	49.7	10.0	806
UI less exercise	45.1	10.2	397
Non-UI more exercise	51.1	8.7	835
Non-UI less exercise	48.2	9.2	365
Main effect of exercise: *F*(1,2399) =82.8 *p* <0.001			
Exercise x UI interaction: *F* (1,2399) = 4.2 *p* < 0.05			
UI more sex	51.4	9.6	357
UI less sex	45.9	10.4	631
Non-UI more sex	53.1	8.0	369
Non-UI less sex	48.0	9.4	539
Main effect of sex: *F*(1,1892) = 145.3 *p* < 0.001)			
Sex x UI interaction: *F* < 1			

The next set of analyses considered the impact of problems associated with UI. Factor analysis showed three categories of problem: Relationships (sex; social life); Work/household/physical activity; and Fatigue/mental health. On the basis of these factor scores the UI sample was split into tertiles in order to provide an initial indication of dose-response. As problems increased, wellbeing decreased (mainly in the last tertile - see Table 5), although the effects were bigger for relationships and fatigue/mental health than the work/household/physical category.

**Table 5:** Wellbeing scores (mean, s.d,) and impact of UI (shown as tertiles, tertile 1 = lowest impact)

Factor	T1	T2	T3	
Relationships	49.7 9.2	51.3 9.5	45.5 10.9	F2,978 = 30.2 *p* < 0.001
Work/household/physical activities	49.9 10.1	48.9 9.5	47.7 10.9	F2,978 = 4.0 *p* <0.05
Mental health	52.4 9.1	49.7 9.7	44.4 10.1	F2,978 = 58.0 *p* < 0.001

Wellbeing also decreased as a function of the degree of embarrassment associated with UI (*F* (2,1200) = 68.7 *p* < 0.001: Never embarrassed: mean = 51.8 s.d. = 9.3; Sometimes embarrassed: mean = 47.6 s.d. = 9.3; Often/All the time: mean = 41.9 s.d. = 11.6). A fourth set of analyses examined when they leaked and the use of pads. Factor analyses (*N* = 1177) showed three categories of leakage: No obvious reason; Cough/sneeze/exercise; and before getting to the toilet. On the basis of these factor scores the UI sample were split into tertiles. An increase in all these types of leakage was associated with reduced wellbeing (see Table 6) as was frequency of wearing pads (*F*(3,1199) = 5.1 *p* <0.005: Never: mean =50.5 s.d. = 9.2; Sometimes: mean = 48.6 s.d. = 10.2; Often/All the time: mean = 47.1 s.d. = 10.5). Frequency of using pads was associated with more severe UI which in turn was associated with lower wellbeing (Light UI: *N* = mean = 49.4 s.d. = 9.5; Medium UI: mean = 44.6 s.d. = 11.4; Severe UI: mean = 42.2 s.d. = 12.7).

**Table 6:** Reason for leakage (shown as tertiles; T1 = not present) and wellbeing (mean, s.d., N)

Factor	T1	T2	T3	
No obvious reason	50.0 9.0	49.0 9.2	44.8 11.5	F2,1174 = 29.9 *p* < 0.001
Cough/sneeze/exercise	48.4 10.7	48.6 10.3	47.0 9.6	F2,1174 = 2.9 *p* < 0.05
Before getting to toilet	48.9 10.5	48.8 9.4	46.2 10.7	F2,1174 = 8.7 *p* <0.001

## Discussion

These results showed that reduced wellbeing is observed in UI. This effect is observed in all aspects of wellbeing and most sub-groups of UI sufferers. Factors which reflect greater severity of UI lead to a greater reduction in wellbeing. Lifestyle influences wellbeing and those with UI who exercise less frequently or have sex infrequently are especially likely to report reduced wellbeing. Wellbeing decreases as a function of the severity of UI and reductions in HRQol. Again, these changes reflect all aspects of wellbeing measured by WEMWBS. Previous analyses [13] showed that when both quality of life (ICIQ-LUTSqol) and symptom severity (ICIQ-UI Short Form) were included in the same regression analysis, the only significant effect on mental well-being was ICIQ-LUTSqol. This shows that UI symptoms do not directly affect mental well-being, but that symptoms influence quality of life, which in turn influences well-being.

This is the first multi-national study which has examined the impact of UI on wellbeing. The sample consisted of women aged 45–60 in four developed countries and this population enables assessment of wellbeing in a group with a busy active life which is likely to be more demanding than that of elderly populations who are often studied in UI research. The present sample consisted of women who largely reported light UI and yet the results show a clear reduction in wellbeing in this group. Those with severe incontinence find it extremely difficult to engage in sex and physical exercise but by sampling those with light UI the present study was able to look at how those activities related to wellbeing in both UI and non-UI groups. Improving wellbeing in those with mild UI is clearly very important and shows that clinical management must address this as well as the issue of leakage. UI is often under reported and community education is desirable as those with mild UI may not see a clinician. It is also important to investigate the extent to which individuals adopt compensatory behaviours which enable them to function and minimize the problems related to UI.

The study has a number of limitations and further research is required to extend the present findings and reduce any potential biases present in this study. The online format of the survey may have excluded certain groups and future research should consider a case-control approach to the comparison of UI and non-UI groups. Type of incontinence (stress, urgency and mixed) is also a factor which can now be assessed using wellbeing outcomes. More specific information about exercise and sexual behaviour is also required, as is information on BMI which may be important in UI and wellbeing. Other psychosocial factors known to influence wellbeing (e.g., personality; stress; social support and coping) should also be measured in order to determine whether the effects of UI and these psychosocial factors have independent, additive or interactive effects on wellbeing. The practical significance of the small difference in wellbeing also needs to be assessed and bench marked against effect sizes seen in other chronic conditions. The WEMWBS was not really designed to determine clinical significance and other measuring instruments should be also used to determine whether any differences are clinically significant.

## Conclusion

In conclusion, the results from the present survey demonstrate that even light UI is associated with lower wellbeing. This appears to be a general problem associated with UI in that it is present in most demographic groups and is observed in all the domains measured by the WEMWBS. Within the UI group the lower wellbeing was related to HRQol and to perceptions of interference with specific activities or the induction of mental health issues. Therapies aimed at increasing wellbeing (e.g., mindfulness; Cognitive Behaviour Therapy) may play a role in the management of UI.
